# It Can’t B: An Unusual Case of Streptococcus agalactiae (Group B) Empyema Necessitans

**DOI:** 10.7759/cureus.54140

**Published:** 2024-02-13

**Authors:** Muhddesa Lakhana, Vivianne Sambour, Daniel Kurbanov

**Affiliations:** 1 Internal Medicine, Mount Sinai South Nassau, Oceanside, USA; 2 Hospital Medicine, Mount Sinai South Nassau, Oceanside, USA; 3 Pulmonary and Critical Care Medicine, Mount Sinai South Nassau, Oceanside, USA

**Keywords:** conservative approach, thoracic radiology, group b streptococcus agalactiae bacteremia, infectious disease pathology, pulmonary disease

## Abstract

Empyema necessitans is characterized by an empyema that extends from the lung pleura into the chest wall and underlying tissue. We present a rare case of *Streptococcus agalactiae* (Group B) empyema necessitans in an adult male. This case highlights the diagnosis and management of empyema necessitans in the modern era.

## Introduction

Empyema necessitans is an empyema that extends from the lung pleural space into the chest wall and overlying tissue. This is a rare complication and is more commonly associated with mycobacterium tuberculosis and actinomyces than with typical community-acquired organisms [1]. Treatment includes antibiotics targeted for the specific bacteria, and in severe cases chest tube placement and often surgical intervention such as decortication of the lung parenchyma. We present a rare case of *Streptococcus agalactiae* (Group B) empyema necessitans in an adult male the management of empyema necessitans in the modern era.

## Case presentation

A 61-year-old male presented to our hospital with acute abdominal pain with hematemesis for one day. Significant history included former tobacco smoking with cessation 30 years ago, throat cancer status post-chemo-radiation, syndrome of inappropriate antidiuretic hormone secretion (SIADH) previously on tolvaptan, and gastroesophageal reflux disease. He reported bloody emesis that started one night prior; associated with sharp, burning epigastric pain and nausea. He also reported one week of right shoulder pain and two days of right anterior “fullness” in his chest. He was taking ibuprofen and was unsure of the dosage, four times daily for three days, for right shoulder pain that started after lifting heavy boxes. There was no history of fevers, chills, trauma, recent illnesses, or any sick contacts.

**Figure 1:**
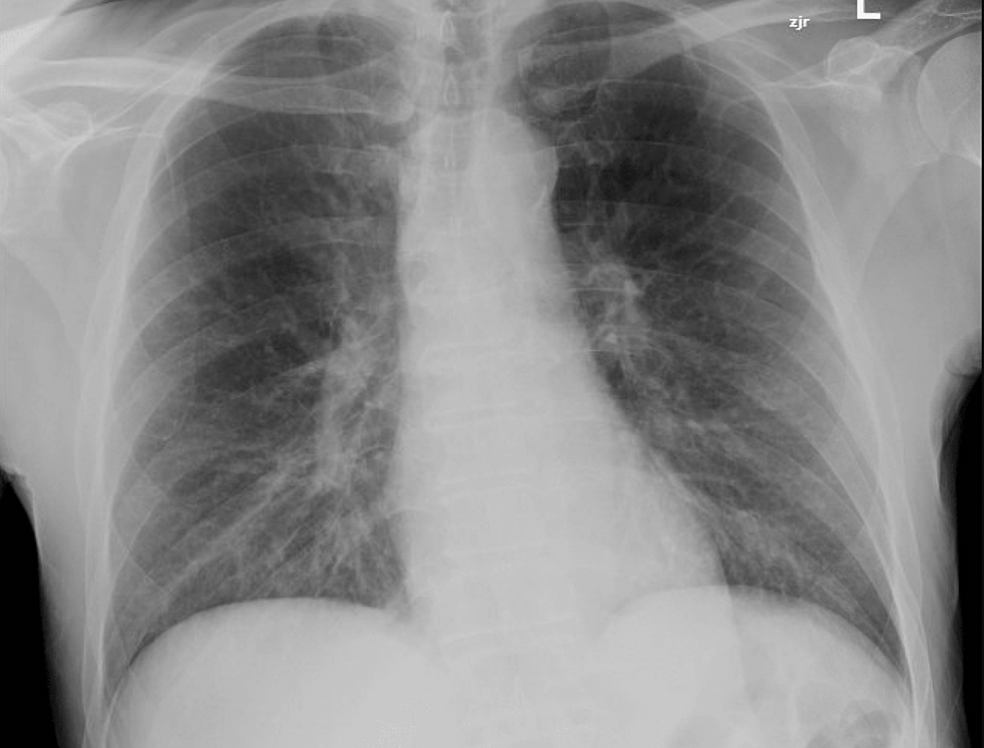
Initial chest radiograph: normal chest radiograph

At presentation, his vitals included an initial blood pressure of 141/62 mmHg, and subsequently 95/69 mmHg. Heart rate was 83 beats a minute. Oxygen saturation was 97% on room air. Temperature 98.4 F. Physical exam revealed a hoarse voice and vesicular breath sounds bilaterally on lung auscultation. Laboratory data were significant for leukocytosis (white blood cell count 17.8 with 90% neutrophils) and hyponatremia (sodium 115 mEq; baseline 125-128 mEq). Portable chest radiographs did not show focal airspace opacities or pleural effusions (Figure 1).

**Figure 2:**
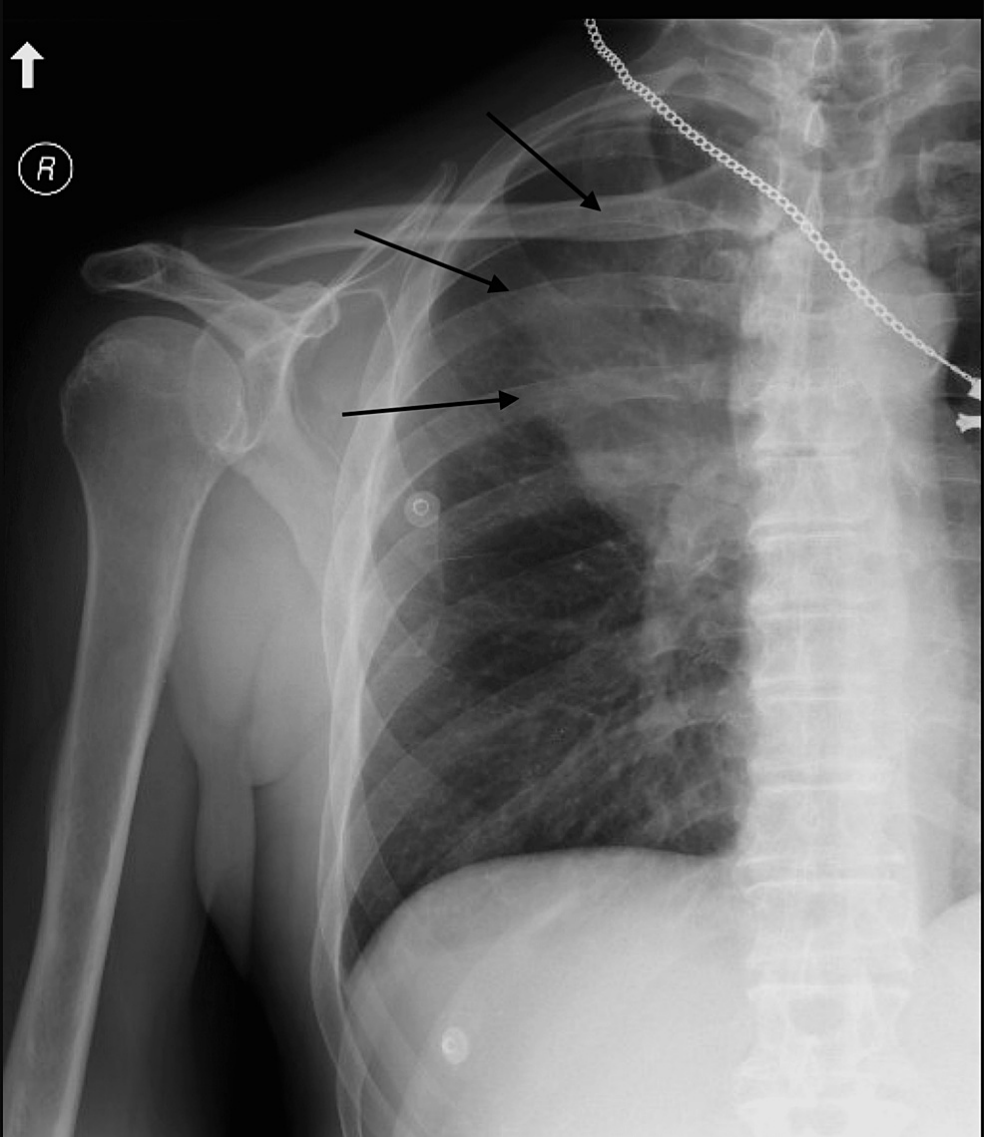
Second radiograph shows a well-defined opacity in the upper zone.

Electrocardiogram revealed a new left bundle branch block which was thought to be the cause of the chest pain initially. During his hospital course, there were no further episodes of hematemesis and nausea improved once hyponatremia was controlled with tolvaptan. Cardiac catheterization revealed mild non-obstructive coronary artery disease and nonischemic cardiomyopathy with an ejection fraction of 35-40%. He continued to have right-sided chest pain and shoulder pain and a right shoulder radiograph was obtained which revealed a lung mass (Figure 2). Mass was new when compared to the initial radiograph taken four days prior.

**Figure 3:**
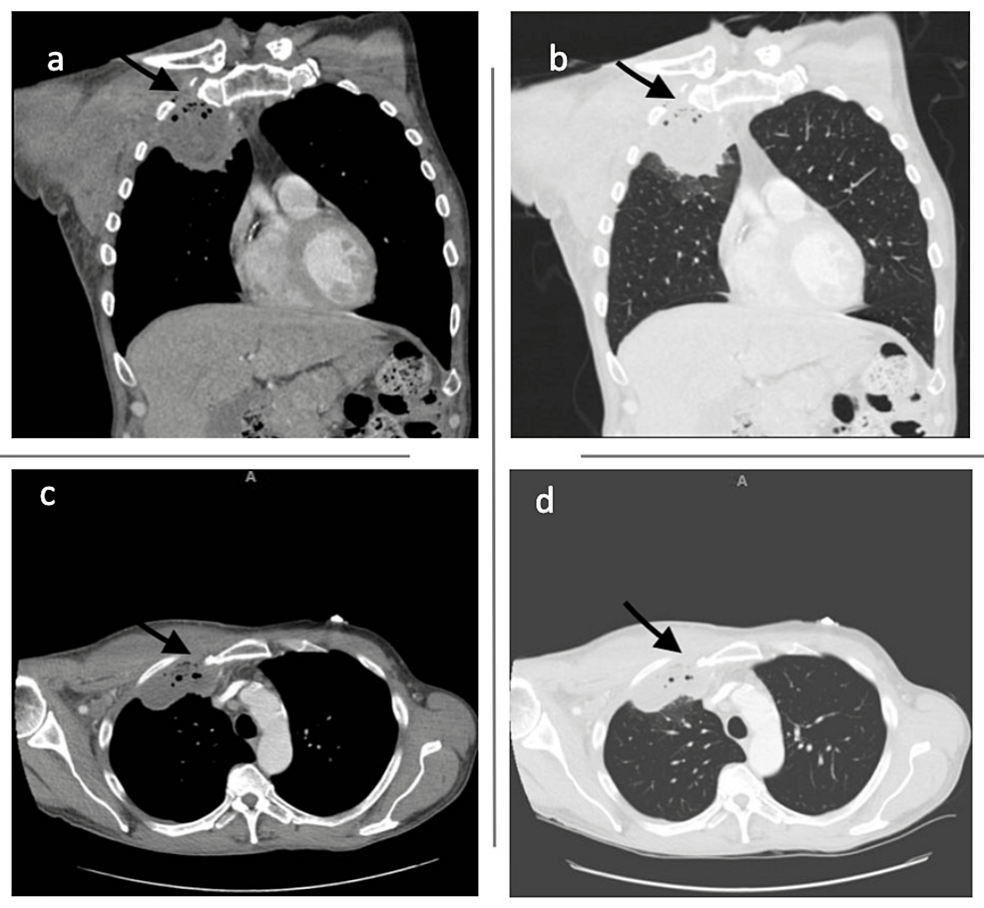
CT chest with IV contrast. Mass is present in the right upper lobe; (a) coronal view; (b) coronal view-lung window; (c) transverse view; (d) transverse view-lung window. CT: computed tomography, IV: intravenous

Further investigation with contrast-enhanced computed tomography (CT) of the chest revealed a 6.5x3.8cm complex right pleural space collection with air foci within the right pectoral muscles (Figures 3a-3d).

**Figure 4:**
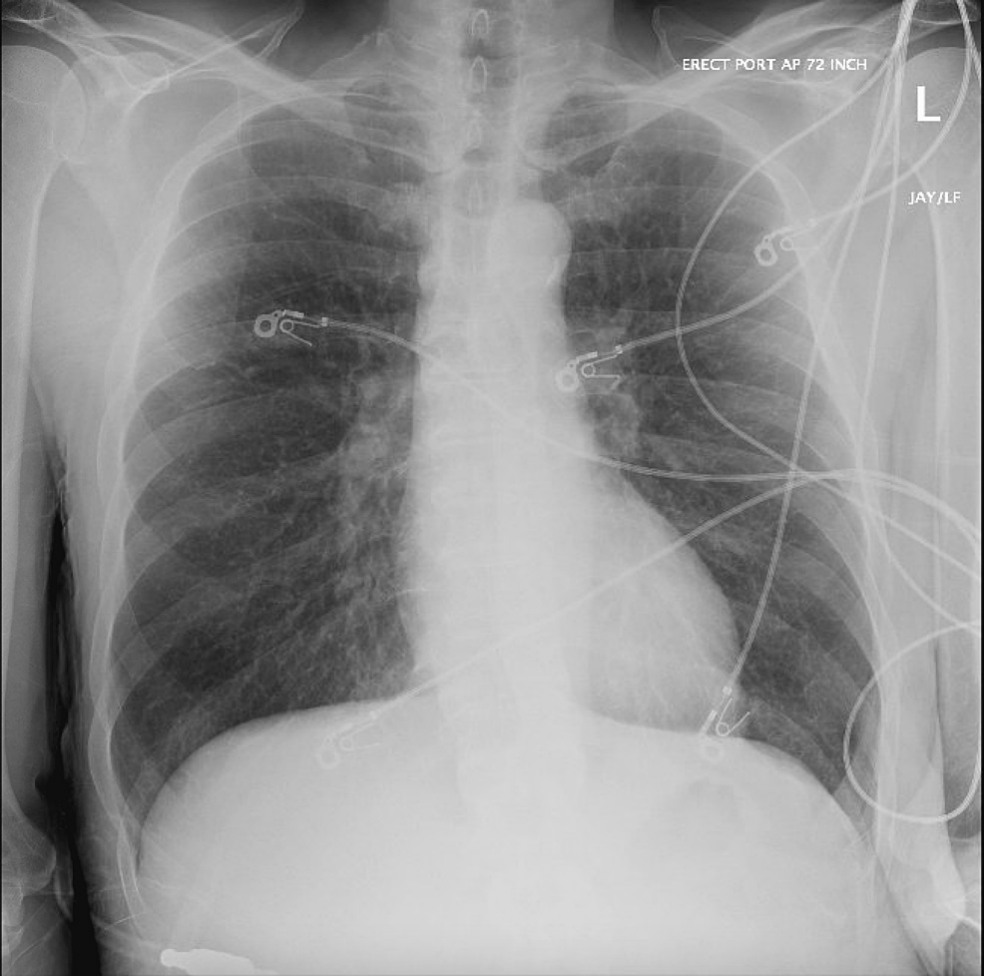
Chest radiograph six months after discharge

CT-guided drainage was performed by interventional radiology, 15 cc of purulent material was removed and a 12 French pigtail catheter was placed. The abscess grew Group B streptococcus (GBS). Pathology revealed bacteria, no evidence of fungal growth, and no evidence of malignant cells. The acid-fast bacillus (AFB) smear was negative for mycobacteria. HIV testing was nonreactive. Initially, he was started on Piperacillin-Tazobactam but was switched to Ampicillin/Sulbactam once sensitivities resulted. A thoracic surgery consultation was requested, and the initial plan was for surgical intervention, however, the patient self-removed the drain and declined surgery [2]. After multidisciplinary discussion involving infectious disease consultants, pulmonologists, and thoracic surgeons. The patient was discharged with a plan for six weeks of Ampicillin/Sulbactam and repeat chest imaging in four weeks. The patient was lost to follow-up and did not get repeat imaging four weeks after discharge. The patient's chest radiograph six months after showed a resolution of the infection (Figure 4).

## Discussion

Empyema necessitans occurs when a pleural space infection decompresses through the visceral pleura and into the chest wall tissue and skin. This is a rare complication and is more commonly associated with mycobacterium tuberculosis and actinomyces than with typical community-acquired organisms. Symptoms include pleuritic chest pain, crepitus, and occasionally a bulging mass in the chest cavity [1]. Initial diagnostic modality is often plain radiograph; however, this may not be reliable in identifying the infection and can be a false negative [2]. In our case, a portable chest X-ray was performed and did not reveal the right upper lobe (RUL) lung process. A more accurate modality includes a CT scan which can help make the diagnosis as well as identify the extent of infection, which in this case extended from the pleura into the right pectoralis muscle.

Obtaining a sample is important because it can help distinguish between an infection and malignancy which is the major differential diagnosis, as well as guide antibiotic selection. Our patient was a former smoker with a history of SIADH and throat cancer increasing the consideration for malignancy. A sample can be obtained through various methods including thoracocentesis, chest tube, endobronchial ultrasound, or navigational bronchoscopy. Our patient underwent CT-guided drainage which allowed for precise drain placement without the requirement for sedation or anesthesia.

Samples obtained should be stained for bacterial, fungal, and mycobacterial organisms. Cytology is helpful to assess for underlying malignancy. For empyema necessitans, treatment includes antibiotics targeted for the specific bacteria, and in severe cases chest tube placement, and often surgical intervention such as decortication of the lung parenchyma [2,3]. Treatment often includes broad-spectrum coverage. Our patient was started on Piperacillin-Tazobactam to cover gram-positive, gram-negative, and anaerobic bacteria. Treatment for GBS is typically penicillin G [4]. The treatment for empyema necessitans is four to six weeks and the duration depends on clinical presentation [5,6].

The most common bacterial infections associated with empyema necessitans include *Mycobacterium tuberculosis* and *Actinomyces*. GBS is typically associated with colonization of the female reproductive tract and neonatal meningitis. The incidence of GBS is increasing among adults with co-morbidities leading to immunocompromise. Although uncommon, it can also cause pneumonia, especially in the elderly [7]. GBS bacteremia is often associated with no identifiable source of infection, and it is predominantly seen in patients with medical co-morbidities [2]. Our patient’s history of throat cancer likely increased his risk for GBS bacteremia and subsequent complications. He had an extension of a pleural empyema into the pectoralis muscle. Our patient was given six weeks of Ampicillin/Sulbactam based on the susceptibility testing.

## Conclusions

This case demonstrates the importance of diagnostic re-evaluation as well as highlighting the possibility of a false negative chest radiograph. In addition, non-surgical management should be considered as effective drainage can obviate the need for more invasive treatment. Empyema necessitans should be considered, even in regions without significant endemic tuberculosis disease, as a potential complication of pleural space infections including parapneumonic effusion and empyema, and may have an insidious presentation as well as atypical risk factors and microbiologic etiology.
